# Effect of Heat Treatment Duration on the Recrystallization and Electrochemical Properties of Cold-Rolled Cantor-Type High-Entropy Alloy

**DOI:** 10.3390/ma18102298

**Published:** 2025-05-15

**Authors:** Byung-Hyun Shin, Jinsurang Lim, Doo-In Kim, Jung-Woo Ok, Seongjun Kim, Jinyong Park, Jonggi Hong, Taekyu Lee, Jang-Hee Yoon, Je In Lee

**Affiliations:** 1 Yeongnam Regional Center, Korea Basic Science Institute, Busan 46742, Republic of Korea; lemonhouse211@kbsi.re.kr (B.-H.S.); jwok@kbsi.re.kr (J.-W.O.); seongjunk@kbsi.re.kr (S.K.); jinyongp@kbsi.re.kr (J.P.); jkhong@kbsi.re.kr (J.H.); taekyulee1@kbsi.re.kr (T.L.); 2 School of Materials Science and Engineering, Pusan National University, Busan 46742, Republic of Korea; wlstn5695@pusan.ac.kr; 3 Innovative Graduate Education Program for Global High-Tech Materials and Parts, Pusan National University, Busan 46241, Republic of Korea; dooin.kim@pusan.ac.kr

**Keywords:** high-entropy alloy, heat treatment time, microstructure, residual stress, electrochemical behavior

## Abstract

High-entropy alloys (HEAs), such as the Cantor alloy, are considered for various structural applications owing to their excellent corrosion resistance and high strength at low temperatures, typically below −70 °C, including cryogenic conditions. However, during metalworking, introducing stresses and grain refinement can reduce the corrosion resistance of HEAs. Recrystallization heat treatment relieves these stresses and homogenizes the grain structure, thereby restoring their corrosion resistance and physical properties. However, inadequate heat treatment can result in a microstructure in which coarse and refined grains coexist; thus, the corrosion resistance is diminished and the physical properties are compromised. Therefore, a proper heat treatment is essential for achieving the desired corrosion resistance and mechanical properties of HEAs. In this study, a cold-rolled high-entropy Cantor alloy was subjected to heat treatment for various durations, and the conditions were analyzed. The microstructure and electrochemical behavior were examined. The results indicated that the grains coarsened after a heat treatment time of 5 min and the residual stresses decreased for 15 min. The potential increased from −0.20 to −0.09 V, whereas the resistance of the passive layer increased from 39 to 56 kΩ. These findings confirm that in the Cantor alloy, residual stress reduction and recrystallization begin after 5 min of heat treatment at 1100 °C, which contributes to the recovery of corrosion resistance. The corrosion resistance of the Cantor alloy can be effectively controlled through heat treatment. This underscores the importance of optimizing the heat treatment process in the manufacturing of Cantor alloys.

## 1. Introduction

Recent advances in manufacturing technology have led to the development of various Fe-based alloys [1,2]. Additionally, as the importance of energy efficiency continues to increase, significant progress is being made in the research on lightweight alloys, such as aluminum [3,4]. The advancements in manufacturing technologies and the diversification of research in the industry have resulted in the development of numerous materials, including a recently developed high-entropy alloy (HEA) [5,6].

Although various new alloys have been introduced, fundamental studies on their electrochemical behavior and heat treatment remain limited. While several recent studies have examined the corrosion behavior of HEAs in acidic or saline environments, the effects of post-deformation processes—such as cold rolling—on corrosion performance have not yet been fully addressed.

Among the various HEAs, Cantor alloy is composed of five elements—Cr, Mn, Fe, Co, and Ni—in equal atomic percentages (20%) [7,8]. High-entropy Cantor alloys were proposed by Cantor and exhibit excellent strength and corrosion resistance [9,10]. Their high strength is attributed to the stabilization of the austenite phase due to Mn and Ni; this stabilization increases the number of twins being formed [11,12]. In metallic materials, an increase in twinning enhances the strength because dislocation movements (strength is increased) and elongation (new slip planes are generated after twinning) are inhibited. Additionally, high-entropy Cantor alloys exhibit remarkable strength at low temperatures owing to the ease of twinning under such conditions [13]. Cantor presented the characteristics and established standards for high-entropy Cantor alloys [7]. Otto analyzed the microstructure of high-entropy Cantor alloys and identified the twinning mechanism [9]. Koch analyzed the effects of nanocrystals on HEAs [8]. Although the physical properties of high-entropy Cantor alloys have been extensively studied, research on their corrosion resistance remains insufficient.

In this context, the present study provides a novel contribution by systematically investigating the microstructural evolution and electrochemical behavior of cold-rolled Cantor-type HEAs subjected to different durations of heat treatment. The combined effects of rolling-induced deformation and subsequent recrystallization on corrosion resistance have not been comprehensively addressed in previous studies, thereby establishing the originality and relevance of this work.

The corrosion resistance of metals is typically evaluated by performing electrochemical behavior analyses or field tests [14,15]. The corrosion resistance of high-entropy Cantor alloy is primarily dependent on chromium (Cr), which forms a passive layer [16,17]. The alloy composition, with elements with 20 at.%, includes a Cr content of 18.5 wt.%. The pitting resistance equivalent number (PREN) of high-entropy Cantor alloy (PREN = wt.% Cr + 3.3 wt.% Mo + 16 wt.% N) is comparable to that of AISI 304 (PREN 18.2–18.5) [18,19]. However, the corrosion resistance of high-entropy Cantor alloy can vary depending on the microstructure and residual stresses resulting from the processing conditions [20,21]. Although the microstructure and corrosion resistance of AISI 304 have been studied, high-entropy Cantor alloys exhibit different microstructural characteristics [22,23], and their corrosion resistance should be further assessed, particularly in relation to microstructural differences.

The aim of this study is to investigate the effects of cold rolling and subsequent heat treatment duration on the microstructural evolution and corrosion resistance of high-entropy Cantor alloys. This work specifically focuses on correlating grain refinement, residual stress relaxation, and passive layer formation with electrochemical behavior in order to guide the design of corrosion-resistant HEAs for practical applications.

High-entropy alloys (HEAs), such as the Cantor alloy, are emerging materials with unique compositional and structural properties. While conventional alloys such as steel and stainless steel are well studied and widely used in industrial applications [3,24], the behavior of HEAs under various processing conditions remains unclear due to limited data on their performance in different manufacturing and service environments [15,25]. In particular, the electrochemical behavior of the high-entropy Cantor alloy after cold rolling has not yet been fully investigated [5]. In addition, its microstructural evolution and corrosion resistance following cold working and subsequent heat treatment remain underexplored. Therefore, this study aims to examine the cold-rolled microstructure, heat treatment effects, and electrochemical properties of the Cantor alloy to assess its potential for commercial applications.

The scope of this study is limited to laboratory-scale conditions. Extensive research is required to optimize the use of the high-entropy Cantor alloy, with a critical focus on the analysis of the corrosion resistance. In this study, the effects of the manufacturing and heat treatment processes on the microstructure and corrosion resistance of the high-entropy Cantor alloy were investigated. The alloy’s microstructure was analyzed using field-emission scanning electron microscopy (FE-SEM) and electron backscatter diffraction (EBSD) after implementing casting and cold-rolling processes. The cold-rolled high-entropy Cantor alloy was subjected to heat treatment for various durations of up to 60 min, and the impact of heat treatment on the microstructure and corrosion resistance was thoroughly evaluated. Microstructural analysis was conducted using FE-SEM and EBSD, whereas the corrosion resistance was assessed by employing electrochemical methods. The electrochemical analysis included open-circuit potential (OCP) and potentiodynamic polarization tests performed by using a potentiostat. The passivation layer properties were evaluated by using the critical pitting temperature (CPT), electrochemical impedance spectroscopy (EIS), and X-ray photoelectron spectroscopy (XPS). The chemical composition of the surface in the depth direction was analyzed using glow-discharge spectroscopy (GDS) at different heat treatment times.

## 2. Materials and Methods

### 2.1. Manufacturing Process

**Figure 1:**
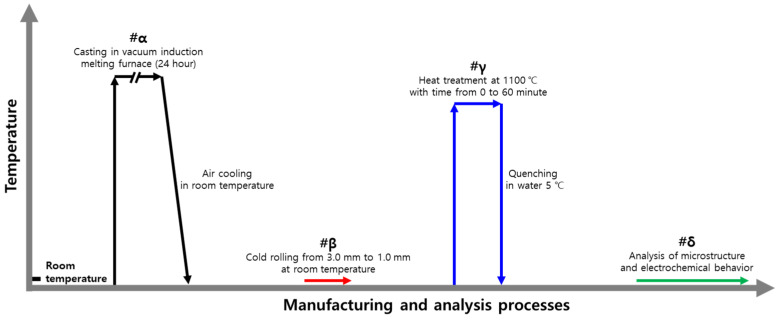
Schematic of the manufacturing and analysis processes for high-entropy Cantor alloy.

The manufacturing and analysis processes for the HEA are illustrated in Figure 1. To control the chemical composition of the HEA, casting (#α, output temperature 1400 °C) was performed in a vacuum induction-melting furnace, yielding a 10 kg slab [6,26].

The molten alloy was poured into an alumina-coated graphite mold (30 cm × 10 cm × 3 cm) under an inert argon atmosphere. Solidification occurred via air cooling (2 °C/s) in ambient air without directional control. To minimize microstructural variation caused by thermal gradients, the specimens were extracted from the central region of the cast slab.

The chemical composition of the slab, measuring 30 cm × 10 cm, was verified using inductively coupled plasma mass spectrometry (Thermo Fisher Scientific, Waltham, MA, USA). Table 1 presents the chemical composition of the HEA.

After casting was performed, the composition-controlled HEA was subjected to cold rolling (#β) at room temperature. The rolling process was conducted in three passes, each with a reduction ratio of 30% to 0.1 mpm (without lubricant, dry condition). Cold rolling reduced the thickness of the HEA from 3.0 ± 0.06 mm to 1.0 ± 0.02 mm [2,27]. The samples were then machined to achieve dimensions of 1.5 mm × 1.5 mm.

### 2.2. Heat Treatment

After being cold-rolled, high-entropy Cantor alloys require heat treatment (#γ) to relieve stress and have a homogenized microstructure. Thus, the rolled HEA was subjected to heat treatment in a box furnace at 1100 °C for durations ranging from 0 to 60 min, followed by quenching in 5 °C water.

Heat treatment durations ranging from 0 to 60 min were selected to observe the key stages of recrystallization and grain growth. This time frame was determined based on preliminary testing, which showed that the most significant microstructural and electrochemical changes occur within the first 60 min. Beyond this duration, the system exhibited only marginal variation, indicating saturation behavior.

The effects of the heat treatment and the microstructural changes after rolling were analyzed using EBSD (SUPRA 40VP system, Zeiss; Oberkochen, Germany). The EBSD scan was performed with a step size of 0.13 µm to accurately resolve the fine grain structures. Recrystallization was identified by conducting an image quality (IQ) analysis, and the average grain size was measured [28,29]. The grain growth orientation was determined using an inverse pole figure (IPF), and the stress distribution was assessed by applying the kernel average misorientation (KAM) mode [30,31].

### 2.3. Electrochemical Behavior

The electrochemical behavior was evaluated by observing the electrical changes in the high-entropy Cantor alloy in an electrolyte solution. A potentiostat (Versa State 4.0; Princeton, NJ, USA) and three-electrode cell were employed for the analysis [32,33]. The three-electrode setup consisted of a working electrode (the specimens), a reference electrode (a saturated calomel electrode), and a counter electrode (a platinum mesh, 20 mm × 20 mm) [34,35]. Prior to testing, the specimen surfaces were mechanically polished and finished to achieve a surface roughness of 100 nm, ensuring uniform electrochemical measurements. The electrochemical behavior was analyzed using the OCP, potentiodynamic polarization testing, and EIS, with the measurement area of the specimens being 1 cm^2^. The electrolyte solution used for the experiments was 3.5 wt.% NaCl, prepared as per ASTM G 61 [19,36]. For the CPT test, a 5.85 wt.% NaCl solution was used in accordance with ASTM G 150-99 [37,38].

The electrochemical series shows the potentials at which pure metals, such as chromium (Cr, −0.91 V), manganese (Mn, −1.18 V), iron (Fe, −0.44 V), cobalt (Co, −0.28 V) and nickel (Ni, −0.25 V), undergo oxidation and reduction. However, because the potentials of alloys cannot be calculated directly, the potentials of HEAs are measured using the open-circuit potential (OCP) [39,40]. In this study, the scan rate for the OCP was 0.5 s, and measurements were performed for up to 3600 s. Potentiodynamic polarization tests were conducted to analyze the corrosion behavior of the material by measuring the change in the current density as a function of the potential [38,41]. The tests on the HEA, after various heat treatment durations, were performed with a voltage ranging from −0.6 to 0.9 V and a scan rate of 0.167 mV/s. EIS was used to measure the change in the resistance over a frequency range of 10^6^–10^−1^ Hz [32,42] to analyze the metal surface.

The CPT was employed to compare the points at which the passivation layer broke down. Although the material used in this study had the same chemical composition as the Cantor alloy, microstructural differences due to the manufacturing processes affected the CPT [38,43]. The CPT test was conducted in a 5.85 wt.% NaCl electrolyte solution at a heating rate of 1 °C/min. The CPT was determined as the temperature at which the current density exceeded 100 µA/cm^2^ for 1 min.

The state of the passivation layer was analyzed using XPS [44,45]. The HEA surface was polished with colloidal silica and cleaned before the analysis. The binding energy was measured using an XPS system (K-Alpha, Thermo Fisher Scientific, Waltham, MA, USA). The analysis was conducted based on the manufacturing process used to observe the effects of annealing. The surface area analyzed by using XPS was 5 mm × 5 mm.

The chemical composition of the surface in the depth direction was analyzed by performing GDS (Horiba Jobin Yvon, JY-10000 RF, Paris, France) at different heat treatment times and a rate of 0.1 nm/s up to a depth of 25 nm. The calculated area was 4 cm^2^. The depth scale in the GDS analysis was calibrated using a SiO_2_ standard of known thickness, and the sputtering rate was determined under the same plasma conditions. The sputtering time was then converted to depth (in nm) to quantify the thickness of the passive and mixed layers.

## 3. Results and Discussion

### 3.1. Microstructure with Manufacturing Process

**Figure 2:**
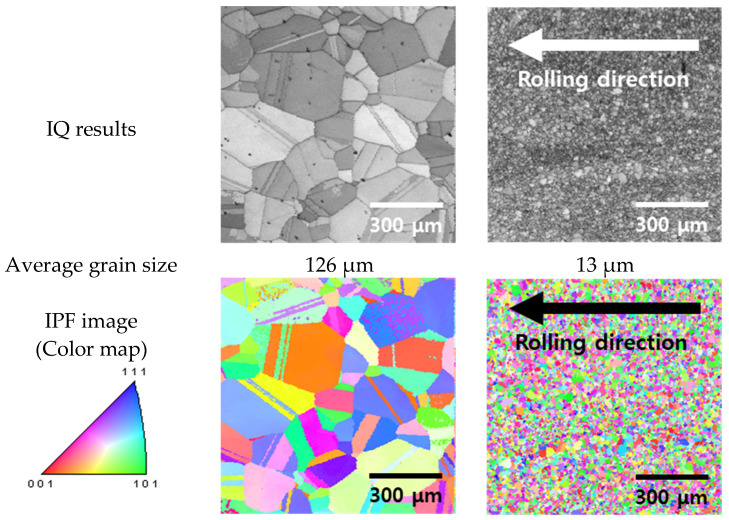

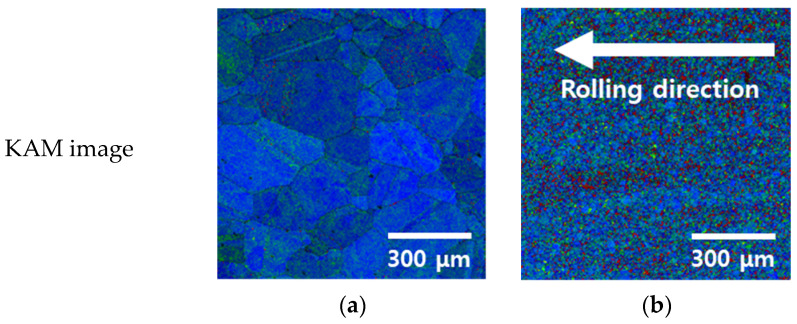
Electron backscatter diffraction (EBSD) image of the manufacturing process for the high-entropy Cantor alloy: (**a**) casting and (**b**) cold rolling. IQ: image quality; IPF: inverse pole figure; KAM: kernel average misorientation.

To manufacture the HEAs as sheets, the microstructural changes after casting and cold rolling were analyzed, as illustrated in Figure 2 [28,31]. The analysis was conducted using EBSD techniques, including IQ, IPF, and KAM. The EBSD analysis revealed that both the cast and rolled microstructures of the HEA formed a face-centered-cubic structure.

The IQ results showed that the cast microstructure of the HEA contained grains that had increased in size to up to 126 μm [24,45] with twinning due to the slow cooling, and numerous twins formed within the grains [11,46]. Additionally, KAM mode analysis indicated the presence of residual stress around the twin boundaries. After being cast, the HEAs were cold-rolled to reduce the thickness from 3.0 to 1.0 mm, resulting in a refined microstructure with an average grain size of 13 μm [24,47]. The grains were refined along the rolling direction, and the residual stress increased in the cold-rolling direction.

The cast microstructures of conventional metals typically exhibit reduced corrosion resistances owing to their inherent heterogeneity, whereas the cold-rolled microstructures exhibit decreased corrosion resistances because of grain refinement and increased residual stresses [48]. Therefore, heat treatment is necessary for enhancing the corrosion resistances of cast and cold-rolled microstructures.

A significant grain size reduction was observed when comparing the as-cast and as-cold-rolled microstructures, as shown in Figure 2. This dramatic change can be attributed to the severe plastic deformation induced by cold rolling, which fragmented the coarse grains and promoted the formation of dislocation structures and sub-grains. Although the high-entropy Cantor alloy possesses inherent thermodynamic grain stability, it remains susceptible to microstructural refinement under high strain, particularly when cold rolled at room temperature. This process results in substantial grain size reduction and aligns with previously reported behaviors of FCC high-entropy alloys subjected to cold work.

The microstructural evolution of the high-entropy Cantor alloy exhibited distinct behavior compared to conventional metals [16,38]. While traditional metals typically undergo moderate grain growth after casting and air cooling, reaching an average grain size of 30 μm, the high-entropy Cantor alloy showed pronounced grain coarsening, with grains growing up to 128 μm and exhibiting predominantly equiaxed morphology. However, after cold rolling from 3.0 to 1.0 mm, the grain size of the Cantor alloy was reduced to below 10 μm, similar to that of conventional cold-rolled metals such as SAF304.

This reduction suggests that the severe plastic deformation during rolling can overcome the thermodynamic grain stability often observed in high-entropy alloys. The grain size change observed is likely associated with deformation-induced phase transformations and dynamic recovery mechanisms, driven by complex multi-elemental interactions and high configurational entropy.

### 3.2. Recrystallization with Heat Treatment Time

The heat treatment of cold-rolled microstructures is essential for relieving residual stress and promoting the recrystallization of refined grains [18]. The cold-rolled high-entropy Cantor alloy can be heat-treated according to specific requirements by controlling the treatment conditions. Therefore, the rolled microstructure of the high-entropy Cantor alloy, characterized by grain refinement and increased residual stress, necessitates heat treatment [21,49]. The microstructure shows stress reduction and recrystallization depending on the heat treatment duration. According to previous studies, the average grain size of SAF304 after cold rolling and recrystallization is from 10 to 25 µm, depending on the processing parameters [25,27].

**Figure 3:**
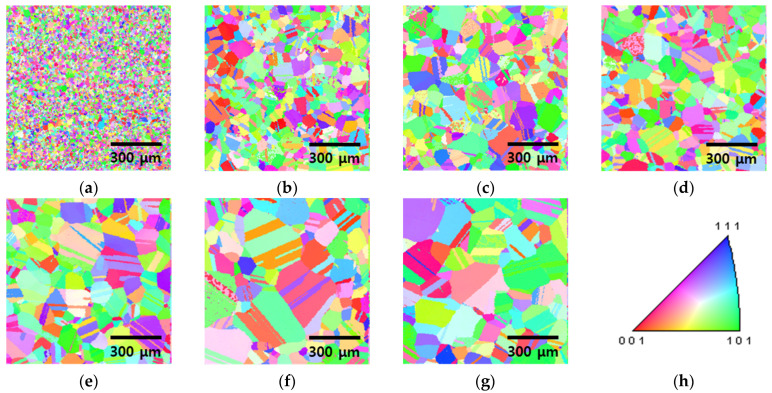
IPF image of EBSD with heat treatment time from 0 to 60 min for the high-entropy Cantor alloy: (**a**) 0 min, before heat treatment, (**b**) 5 min, (**c**) 10 min, (**d**) 15 min, (**e**) 20 min, (**f**) 30 min, and (**g**) 60 min, and (**h**) IPF color map.

**Figure 4:**
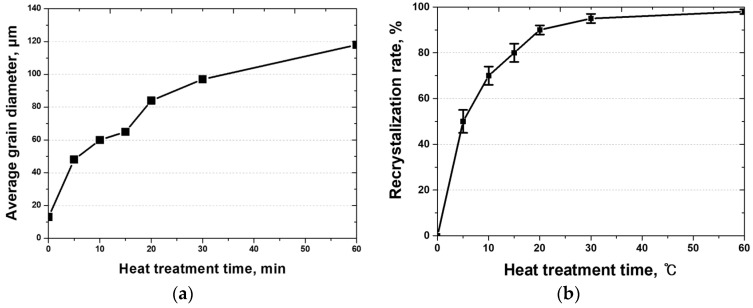
EBSD analysis of the cold-rolled high-entropy Cantor alloy heat-treated at 1100 °C for durations ranging from 0 to 60 min: (**a**) average grain size, and (**b**) recrystallized fraction.

The microstructural evolution of the high-entropy Cantor alloy during heat treatment was analyzed using EBSD, as shown in Figure 3 and Figure 4 [11]. Cold rolling produced fine grains averaging 13 μm, which began to recrystallize after 5 min of heat treatment, increasing the grain size to 48 μm. The grains continued to grow with increasing heat treatment time, reaching 118 μm after 60 min. This microstructural restoration through recrystallization influences both strength and corrosion resistance, where finer grains hinder dislocation motion but may reduce corrosion resistance due to a higher grain boundary density [5,45].

For comparison, the cast HEA, which solidified under air cooling, exhibited a grain size of 126 μm, indicating that the grain size after 60 min of heat treatment approaches the as-cast condition. Changes in residual stress during this process were confirmed by Kernel Average Misorientation (KAM) analysis.

In contrast, conventional alloys such as SAF304 typically exhibit finer grains (~30 μm) even after solution treatment at 1100 °C [25,27], demonstrating the unique grain growth behavior of high-entropy Cantor alloys.

Specifically, the average grain sizes of 13 μm (cold-rolled), 48 μm (5 min), and 118 μm (60 min) were measured in the regions where recrystallization occurred.

**Figure 5:**
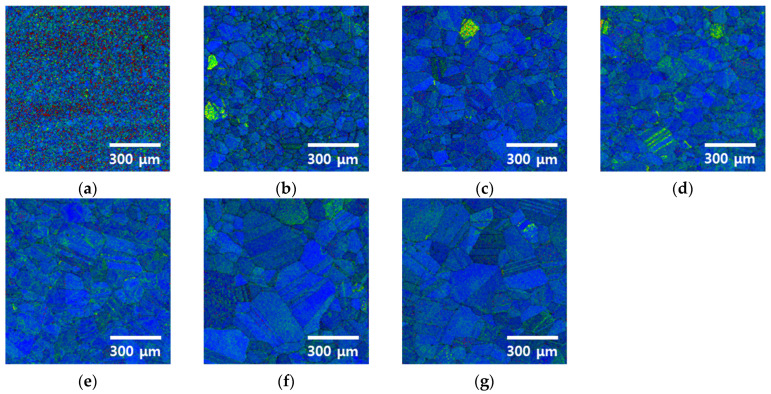
KAM image of EBSD with heat treatment time from 0 to 60 min for the high-entropy Cantor alloy: (**a**) 0 min, before heat treatment, (**b**) 5 min, (**c**) 10 min, (**d**) 15 min, (**e**) 20 min, (**f**) 30 min, and (**g**) 60 min.

The variation in the residual stress with the heat treatment time was assessed using the KAM mode, as shown in Figure 5. In the KAM mode, the fine microstructures formed after rolling exhibit high stress (red dots) and low stress (green dots), which represent the residual stress induced by cold rolling [49]. This is a common characteristic of cold-rolled microstructures. After 5 min of heat treatment, the overall stress decreases, although some residual stress remains in certain grains. As the heat treatment time increases, grain coarsening is observed, and the residual stress within the grains decreases. Within the coarsened grains, residual stress varies owing to twinning.

Residual stress was assessed using Kernel Average Misorientation (KAM) mapping obtained from EBSD analysis. KAM reflects local lattice distortion and is widely used as an indicator of residual plastic strain. A significant decrease in the average KAM value was observed after heat treatment, indicating effective stress relief in the microstructure.

Heat treatment after cold rolling removes the residual stress in the HEA and facilitates recrystallization and grain growth. After cold rolling is employed, the residual stress forms along the cold-rolling direction, and this stress is reduced with 5 min of heat treatment at 1100 °C. Although the stress decreases within 5 min, some residual stress remains, and a steady reduction is observed as the heat treatment time increases. The increased heat treatment time leads to grain coarsening, with grain sizes increasing from 13 to 118 μm. Because the grain size influences dislocation movements, coarser grains result in lower strength, whereas finer grains increase the number of grain boundaries, thereby reducing the corrosion resistance. Therefore, grain coarsening due to the increased heat treatment time for the HEA affects both the material strength and corrosion resistance, and needs to be carefully controlled.

### 3.3. Electrochemical Behavior with Heat Treatment Time

**Figure 6:**
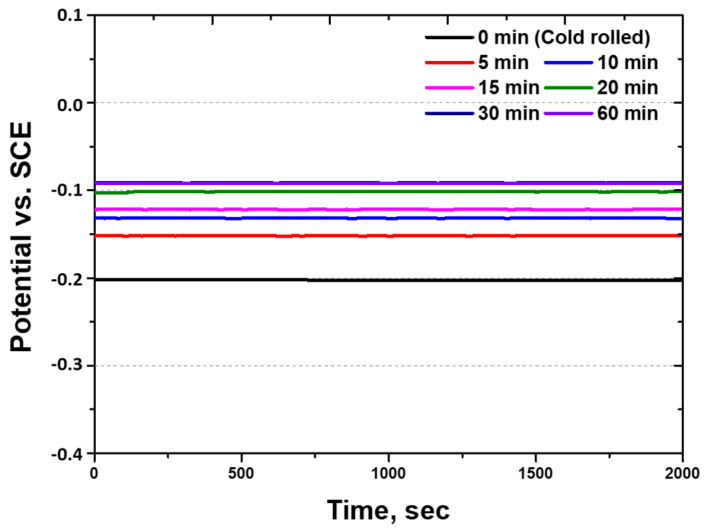
Potential (V) vs. time (s) curve: open-circuit-potential results with heat treatment time of 60 min for cold-rolled high-entropy Cantor alloy in 3.5 wt.% NaCl electrolyte solution. SCE: saturated calomel electrode.

The potentials of pure metals can be calculated using the galvanic potential method with electron and Gibbs free energies [5,6]. However, alloys exhibit various potentials owing to factors such as grain size and galvanic reactions; therefore, the potential is measured using the OCP [50,51]. The potential of the cold-rolled HEA was measured at different heat treatment times, and the results are presented in Figure 6. The potential varies with the heat treatment time. The cold-rolled HEA exhibits the lowest potential of −0.20 V, which increases to −0.09 V after 60 min of heat treatment.

As the heat treatment time increases, the reduction in the residual stress and the coarsening of grains increase the potential, which, in turn, decreases the corrosion reaction [33,42]. Additionally, the potential of the rolled HEA is found to be higher (−0.20 V) than that of Cr (−0.56 V), Mn (−1.18 V), Fe (−0.44 V), Co (−0.28 V), and Ni (−0.25 V). The high potential of the high-entropy Cantor alloy is attributed to the passivation layer formed by Cr, which exhibits characteristics similar to those of stainless steel. The oxide layer formed by Cr contributes to the formation of a uniform layer that increases the potential and corrosion resistance of the high-entropy Cantor alloy.

**Figure 7:**
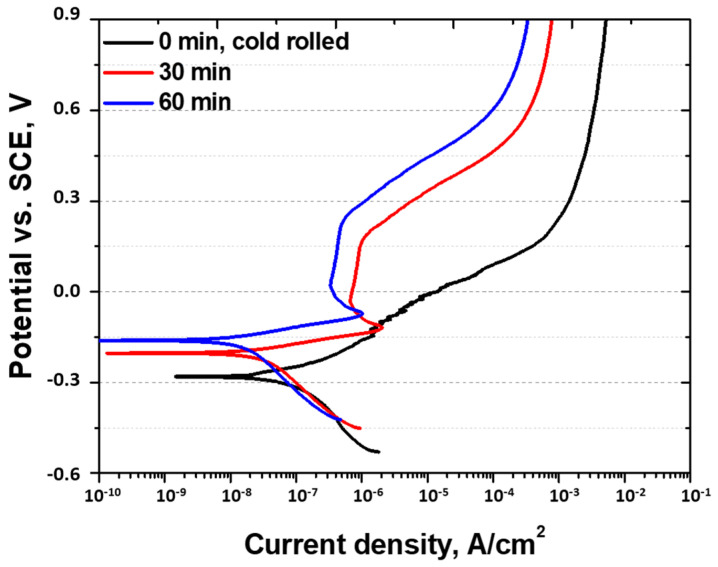
Potential (V) vs. current density (A/cm^2^): potentiodynamic polarization curve with heat treatment time of 60 min for cold-rolled high-entropy Cantor alloy in 3.5 wt.% NaCl electrolyte solution.

Potentiodynamic polarization tests were conducted to measure the variations in the current density with the voltage to assess the corrosion behavior of metals. Figure 7 and Table 2 present the potentiodynamic polarization curves and key values of the high-entropy Cantor alloy after cold rolling and heat treatment. In the active polarization region, the potential (Ecorr) increases from −0.29 to −0.15 V with an increasing heat treatment time, indicating a transition to uniform corrosion after passivation. The current density (Icorr), which indicates the corrosion rate, decreases from 30 × 10^−8^ to 5 × 10^−8^ A/cm^2^, reflecting improved corrosion resistance. Moreover, an increase in the current density is observed after passivation, demonstrating the functionality of the passivation layer in the HEA. As the heat treatment time increases to 60 min, the potential increases from −0.06 to 0.28 V.

The corrosion behavior of the high-entropy Cantor alloy is similar to that of AISI304, which has been previously studied [21,52]. The corrosion behavior of stainless steel is enhanced because of the formation of a passivation layer due to the Cr oxide layer, and the high-entropy Cantor alloy exhibits the same behavior. With 18.5 wt.% Cr, the high-entropy Cantor alloy has the potential to form an excellent passivation layer.

**Figure 8:**
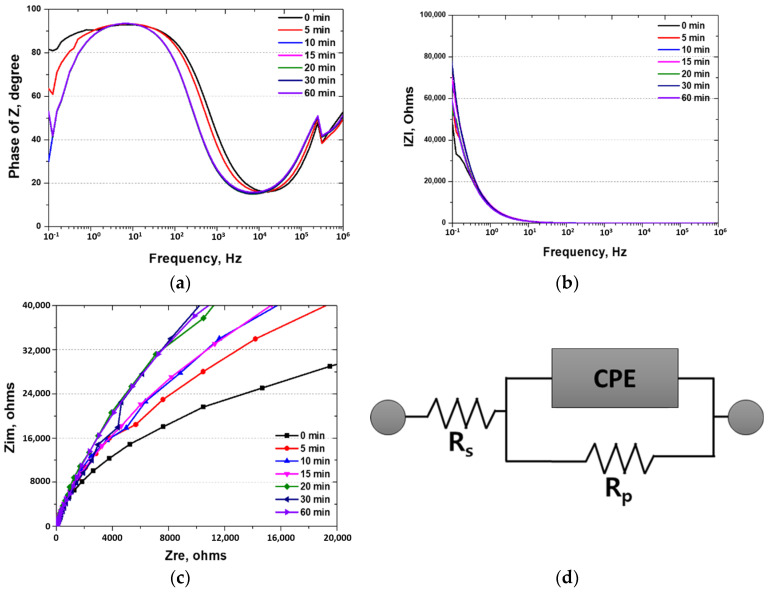
Electrochemical impedance spectroscopy (EIS) results with heat treatment time for high-entropy Cantor alloy in 3.5 wt.% NaCl electrolyte solution. (**a**) Frequency (Hz) vs. phase of Z (resistance degree of phase (°) with frequency); Bode plot. (**b**) Frequency (Hz) vs. lZl (resistance with frequency (ohms)); Bode plot. (**c**) Zre (resistance real (ohms)) vs. Zim (resistance image (ohms)); Nyquist plot. (**d**) Circuit of EIS.

The EIS spectra were further analyzed using both Bode and Nyquist plots, as presented in Figure 8. In the Nyquist plot, the semicircle diameter increases significantly with heat treatment time, indicating a progressive increase in charge transfer resistance (R_ct_), which reflects a more stable and protective passive film. Similarly, the Bode plot shows a notable rise in impedance modulus at low frequencies and a phase angle approaching −80°, indicative of enhanced capacitive behavior and improved surface uniformity.

To quantitatively interpret these trends, an equivalent circuit model consisting of R_s_ (solution resistance), R_ct_ (charge transfer resistance), and a constant phase element (CPE) was used. The fitted parameters, summarized in Table 3, confirm that R_ct_ increases from 39 kΩ to 59 kΩ with increasing heat treatment time. This trend validates the formation of a thicker and more protective Cr-rich passive layer, as also observed in the GDS analysis. These findings confirm that heat treatment enhances the electrochemical stability and corrosion resistance of the high-entropy Cantor alloy.

**Figure 9:**
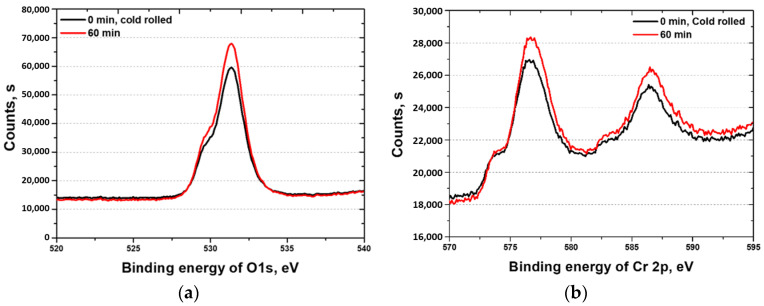
Counts (s) vs. binding energy (eV) curve; XPS results for the different manufacturing processes for the high-entropy Cantor alloy: (**a**) Cr 2p3; (**b**) O 1s.

XPS was used to analyze the surface composition of the HEA, and the results are presented in Figure 9. The main components of the HEA surface are Cr and O, with Cr existing in the Cr^2+^ state and O in the O^2−^ state [44]. The XPS results suggest the formation of a Cr_2_O_3_ passivation layer, which is consistent with the passivation layers observed in stainless steel. As the heat treatment time increases, the oxide layer on the surface also increases, with a corresponding rise in the Cr ratio, indicating an increase in Cr_2_O_3_ [21]. Thus, the increase in the heat treatment time signifies the strengthening of the passivation layer; this is consistent with the EIS results. Consequently, the corrosion resistance of the HEA is protected by the passivation layer, which is a Cr_2_O_3_ layer formed by Cr.

**Figure 10:**
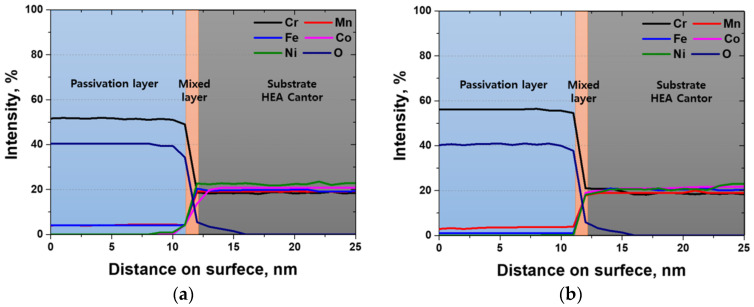
Glow discharge spectroscopy results of Cr, Mn, Fe, Co, Ni, and, O with heat treatment on high-entropy Cantor alloy: (**a**) heat treatment time of 0 min (cold-rolled) and (**b**) heat treatment time of 60 min.

GDS is a technique in which plasma is utilized to analyze surface components and is suitable for analyzing oxide layers. The main alloy components of the HEA were analyzed using GDS, and the results are shown in Figure 10. According to the GDS analysis, a passivation layer with a thickness of 11 nm is uniformly formed on the surface, along with a 1 nm thick mixed layer and the base structure. Among the major components, changes in the Cr and Fe contents are observed at the surface. After heat treatment, the Cr content increases and the Fe content decreases at the surface. This difference affects the stability of the passive layer. An uneven passivation layer reduces corrosion resistance, whereas a uniform passivation layer improves it. Therefore, the reduced concentration of Fe on the surface after heat treatment contributes to the formation of a uniform passivation layer, primarily composed of Cr_2_O_3_ with minor amounts of Fe oxides, thereby enhancing the corrosion resistance.

**Figure 11:**
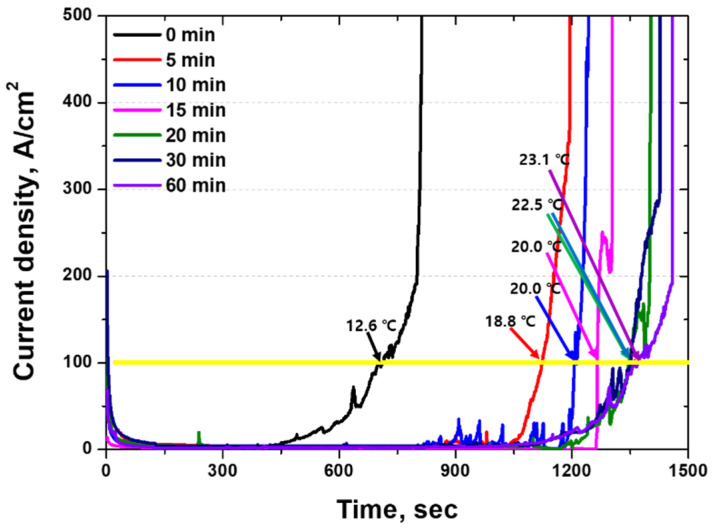
Current density (µA/cm^2^) vs. time (s) curve: heat treatment time for high-entropy Cantor alloy in 5.85 wt.% NaCl electrolyte solution (yellow line: current density at which the CPT is determined).

The CPT test is suitable for evaluating the corrosion resistance of materials with a passivation layer because it is conducted to determine the point at which the passivation layer is destroyed [38,53]. CPT tests were conducted to evaluate the passivation layer of the HEA, and the results are depicted in Figure 11. Changes in the CPT with respect to the heat treatment temperature are observed after rolling. As the heat treatment time increases, the CPT increases from 12.6 to 23.1 °C. This increase is attributed to the recovery of the passivation layer, indicating that the corrosion resistance of the HEA is determined based on the performance of the passivation layer.

**Figure 12:**
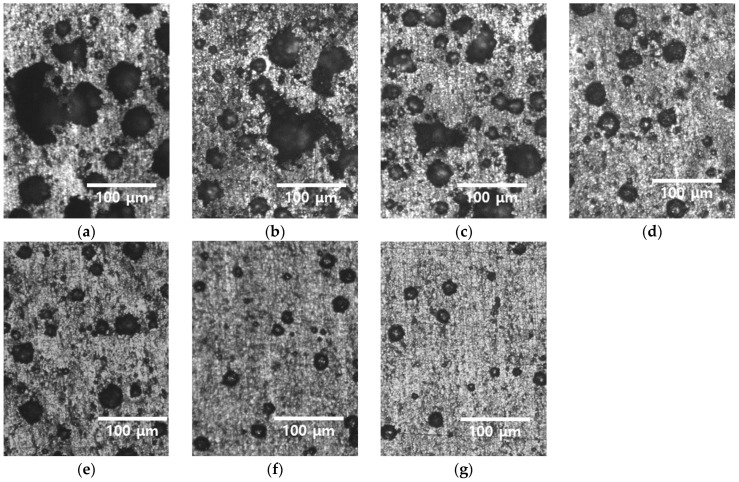
Pitting morphology (black site) with heat treatment time for cold-rolled high-entropy Cantor alloy: (**a**) 0 min, (**b**) 5 min, (**c**) 10 min, (**d**), 15 min, (**e**) 20 min, (**f**) 30 min, and (**g**) 60 min.

**Figure 13:**
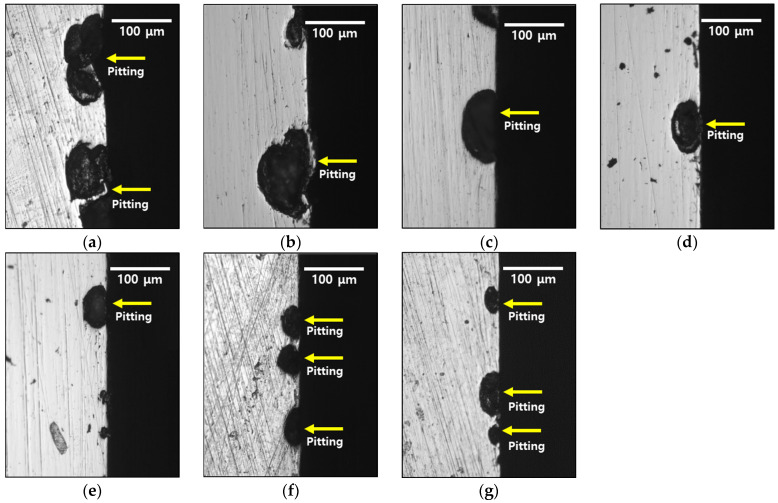
Cross-section image with heat treatment time for cold-rolled high-entropy Cantor alloy: (**a**) 0 min, (**b**) 5 min, (**c**) 10 min, (**d**), 15 min, (**e**) 20 min, (**f**) 30 min, and (**g**) 60 min (yellow arrow: pitting morphology).

After the potentiodynamic polarization tests were conducted, the morphology of the corrosion was examined. The corrosion patterns for different heat treatment times are illustrated in Figure 12 (corrosion image of the surface) and Figure 13 (corrosion image of the cross-section). Corrosion is represented by black spots. In the cold-rolled HEA, corrosion appears as coarse features greater than 50 μm. As the heat treatment progresses, both the number and size of these features decrease. Analysis of the corrosion patterns in the cross-section reveals two major characteristics: (1) merging of corrosion pits owing to growth, and (2) formation of new pits within existing pits.

The corrosion behavior of the HEA is similar to that of stainless steel. This similarity is attributed to the improved corrosion resistance resulting from the passivation layer formed by the 20 at.% (18.5 wt.%) Cr present in the HEA [38,50]. The PREN of the HEA, calculated as PREN = wt.% Cr + 3.3 wt.% Mo + 16 wt.% N, is 18.5, which is equivalent to that of AISI304 (PREN = 18.5) [36]. The corrosion behavior of the HEA is similar to that of AISI304, but its corrosion resistance appears to be superior.

The location of pit initiation in the high-entropy Cantor alloy correlates strongly with the grain structure, particularly the grain boundaries. As observed in Figure 12 and Figure 13, pits predominantly form along grain boundaries rather than within grain interiors. This behavior is especially evident in specimens with smaller grain sizes and high residual stress.

According to classical pitting theory, grain boundaries act as preferential sites for passive film breakdown due to local chemical inhomogeneity, higher energy states, and stress concentration. The susceptibility to grain-boundary-related pitting can be explained by the following relationship between grain size (d) and grain boundary area per unit volume: $S_{Grain\;boundary}\propto \frac{1}{d}$.

As the grain size decreases, the total grain boundary area increases, thereby increasing the number of potential pit initiation sites. In the cold-rolled condition (average grain size ~13 μm), the alloy showed higher pitting density compared to the heat-treated specimen (grain size ~118 μm), suggesting that grain growth during heat treatment reduces the susceptibility to pitting by decreasing grain boundary density.

Therefore, optimizing grain size through appropriate heat treatment is critical not only for improving mechanical properties but also for enhancing corrosion resistance by minimizing grain boundary-related passive film breakdown.

After being cold-rolled, the HEA exhibits a refined microstructure with a grain size of 13 µm and residual stress. The changes in the grain size and residual stress after heat treatment at 1100 °C were evaluated. Consistent with existing theories, the residual stress rapidly decreases within 5 min, whereas grain growth continues for over 60 min. The grain size increases from 13 to 118 μm, and the residual stress overall decreases. The reduction in the residual stress and the increase in the grain size after heat treatment enhance the passivation layer, which improves corrosion resistance.

Metals, such as stainless steel, aluminum alloys, and titanium, are affected by various factors during processing, and the electrochemical properties of the high-entropy Cantor alloy depend on the rolling and heat treatment times. The primary factors influencing the electrochemical properties of HEAs are the residual stress and grain size. The residual stress and fine grains weaken the passive layer, which reduces corrosion resistance. Therefore, an appropriate heat treatment is necessary to achieve the corrosion resistance of HEA after manufacturing. For high-entropy Cantor alloys, heat treatment for a minimum of 5 min is required, and the reductions in the stress after 15 min are not significant. As grain growth continues to enhance corrosion resistance, selecting an appropriate heat treatment time by considering both the strength and corrosion resistance is important.

## 4. Conclusions

An HEA (Cantor) was subjected to cold rolling and subsequent heat treatment to analyze the microstructural evolution and changes in the corrosion resistance. The following conclusions were drawn:

1. The as-cast HEA exhibited coarse grains measuring 126 μm and significant grain growth owing to slow cooling in air. Subsequent rolling decreased the grain size to 13 μm and introduced residual stress along the rolling direction. This behavior mirrored those of conventional metals, demonstrating that HEAs exhibit similar microstructural trends upon casting and rolling.
2. Heat treatment at 1100 °C induced significant changes in the microstructure of the rolled HEA. The residual stresses decreased prior to grain growth, diminished within 5 min, and vanished within 15 min as the grains continued to grow. The grain size increased from 13 to 48 μm after 5 min and further to 118 μm after 60 min. These results reveal increased grain growth compared with that in conventional alloys, such as stainless steel. Because of the high entropy of the alloy, grain growth was more easily facilitated than in conventional metals, such as stainless steel and pure copper, and this aided in the reduction in residual stresses. Consequently, the high-entropy Cantor alloy required more stringent heat treatment conditions than traditional metals.
3. The corrosion resistance of the high-entropy Cantor alloy was influenced by the heat treatment process conducted at 1100 °C for durations ranging from 0 to 60 min. The rolled microstructure exhibited the lowest corrosion resistance, which improved as the grains grew in size. The OCP increased from −0.20 to −0.09 V. Potentiodynamic polarization tests confirmed the enhancement in the corrosion resistance due to heat treatment, with passivation occurring after active polarization. The performance of the passivation layer was further analyzed using the CPT, which increased from 12.6 to 23.1 °C. The state of the passivation layer was verified by performing EIS and XPS, which revealed that the layer primarily consisted of Cr_2_O_3_. Cr contributed to the formation of the passivation layer in the high-entropy Cantor alloy, which is important for corrosion resistance.
4. The passivation layer in the high-entropy Cantor alloy, which was composed of Cr_2_O_3_, played an important role in determining the corrosion resistance. The high-entropy characteristics of the alloy enabled grain growth during the heat treatment. Thus, controlling grain growth through heat treatment is essential because the resultant microstructure significantly impacts the corrosion resistance. As high-entropy Cantor alloys are sensitive to heat treatments, heat treatment processes must be tailored to achieve the desired physical properties, which, in turn, affect corrosion resistance.

## Figures and Tables

**Table 1:** Chemical composition of high-entropy Cantor alloy by ICP-MS.

	Cr	Mn	Fe	Co	Ni	C	O	N	P
at.%	20.0 ± 0.02	20.0 ± 0.02	20.0 ± 0.01	20.0 ± 0.01	20.0 ± 0.01	0.05	0.0004	0.0004	0.0002
wt.%	18.5 ± 0.01	19.6 ± 0.01	19.9 ± 0.01	21.1 ± 0.01	20.9 ± 0.01	0.01	0.0001	0.0001	0.0001

**Table 2:** Major value on potentiodynamic polarization curve with heat treatment time of 60 min for cold-rolled high-entropy Cantor alloy in 3.5 wt.% NaCl electrolyte solution.

	E_corr_	I_corr_	E_pit_
0 min	−0.29 V	30 × 10^−8^ A/cm^2^	−0.06 V
30 min	0.21 V	10 × 10^−8^ A/cm^2^	0.19 V
60 min	−0.15 V	5 × 10^−8^ A/cm^2^	0.28 V

**Table 3:** Key values of EIS with heat treatment time for cold-rolled high-entropy Cantor alloy in 3.5 wt.% NaCl electrolyte solution.

Condition	R_s_	CPE		R_p_
		*n*	P	
0 min	7.1 ohms	0.68	5.7 × 10^4^	39 kohms
5 min	7.2 ohms	0.71	6.9 × 10^4^	49 kohms
10 min	7.1 ohms	0.71	7.1 × 10^4^	51 kohms
15 min	7.2 ohms	0.72	7.2 × 10^4^	52 kohms
20 min	7.2 ohms	0.72	7.3 × 10^4^	53 kohms
30 min	7.2 ohms	0.72	7.5 × 10^4^	54 kohms
60 min	7.2 ohms	0.72	7.8 × 10^4^	56 kohms

## Data Availability

The original contributions presented in this study are included in this article. Further inquiries can be directed to the corresponding authors.
